# Assessing the effect of physical differences in the articulation of consonants and vowels on audiovisual temporal perception

**DOI:** 10.3389/fnint.2012.00071

**Published:** 2012-10-01

**Authors:** Argiro Vatakis, Petros Maragos, Isidoros Rodomagoulakis, Charles Spence

**Affiliations:** ^1^Cognitive Systems Research InstituteAthens, Greece; ^2^Computer Vision, Speech Communication and Signal Processing Group, National Technical University of AthensAthens, Greece; ^3^Crossmodal Research Laboratory, Department of Experimental PsychologyUniversity of Oxford, UK

**Keywords:** temporal perception, TOJs, articulatory features, speech, audiovisual, signal saliency, attentional modeling

## Abstract

We investigated how the physical differences associated with the articulation of speech affect the temporal aspects of audiovisual speech perception. Video clips of consonants and vowels uttered by three different speakers were presented. The video clips were analyzed using an auditory-visual signal saliency model in order to compare signal saliency and behavioral data. Participants made temporal order judgments (TOJs) regarding which speech-stream (auditory or visual) had been presented first. The sensitivity of participants' TOJs and the point of subjective simultaneity (PSS) were analyzed as a function of the place, manner of articulation, and voicing for consonants, and the height/backness of the tongue and lip-roundedness for vowels. We expected that in the case of the place of articulation and roundedness, where the visual-speech signal is more salient, temporal perception of speech would be modulated by the visual-speech signal. No such effect was expected for the manner of articulation or height. The results demonstrate that for place and manner of articulation, participants' temporal percept was affected (although not always significantly) by highly-salient speech-signals with the visual-signals requiring smaller visual-leads at the PSS. This was not the case when height was evaluated. These findings suggest that in the case of audiovisual speech perception, a highly salient visual-speech signal may lead to higher probabilities regarding the identity of the auditory-signal that modulate the temporal window of multisensory integration of the speech-stimulus.

## Introduction

The optimal perception (i.e., the successful perception) of speech signals requires the contribution of both visual (i.e., articulatory gestures) and auditory inputs, with the visual signal often providing information that is complementary to that provided by the auditory signal (e.g., Sumby and Pollack, 1954; Erber, 1975; McGrath and Summerfield, 1985; Summerfield, 1987; Reisberg et al., 1987; Arnold and Hill, 2001; Davis and Kim, 2004; Ross et al., 2007; Arnal et al., 2009). Speech intelligibility has been shown to be fairly robust under conditions where a time discrepancy and/or a spatial displacement has been introduced between the auditory and/or visual stream of a given speech signal (e.g., Munhall et al., 1996; Jones and Jarick, 2006). The present study focuses on the former case, where a signal delay (either auditory or visual) is present in a congruent audiovisual speech stream. Such delays occur frequently in everyday life as the by-product of poor transmission rates often found in broadcasting or sensory processing delays (e.g., Spence and Squire, 2003; Vatakis and Spence, 2006a).

**Figure 1:**
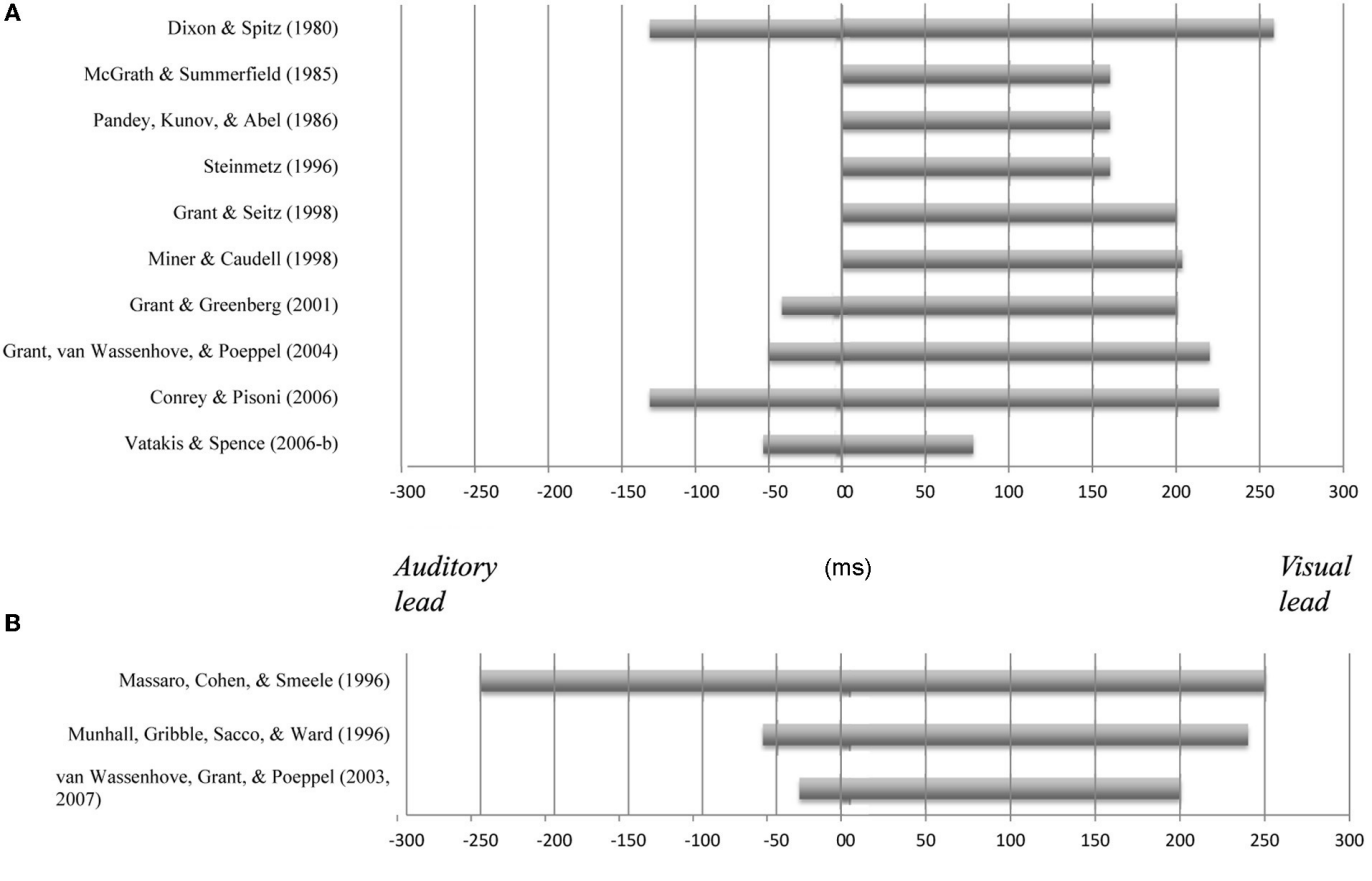
**Variability in the temporal window of multisensory integration observed in previous studies using continuous audiovisual speech stimuli and a variety of different response measures (including identification tasks, simultaneity judgment task, etc.; (A) and brief speech tokens in McGurk-type presentations (B)**.

In order to understand how audiovisual speech perception is affected by the introduction of temporal asynchronies, researchers have evaluated the limits of the temporal window of audiovisual integration (i.e., the interval in which no temporal discrepancy between the signals is perceived; outside of this window, audiovisual stimuli are perceived as being desynchronized) and the specific factors that modulate the width of this temporal window (e.g., Vatakis and Spence, 2007, 2010). One of the first studies to investigate the temporal perception of speech stimuli was reported by Dixon and Spitz (1980). Participants in their study had to monitor a video of a man reading prose that started in synchrony and was gradually desynchronized at a rate of 51 ms/s (up to a maximum asynchrony of 500 ms) with either the auditory or visual stream leading. The participants had to respond as soon as they detected the asynchrony in the video. Dixon and Spitz reported that the auditory stream had to lag the visual stream by an average of 258 ms or lead by 131 ms before the asynchrony in the speech signal became noticeable (see also Conrey and Pisoni, 2003, 2006, for similar results using a simultaneity judgment, SJ, task; i.e., participants had to report whether the stimuli were synchronous or asynchronous). More recently, Grant et al. (2004), using a two-interval forced choice adaptive procedure, reported that participants in their study only noticed the asynchrony in audiovisual sentences when the auditory-speech led the visual-speech signal by at least 50 ms or else lagged by 220 ms or more (see also Grant and Seitz, 1998; Miner and Caudell, 1998; Grant and Greenberg, 2001). Meanwhile, McGrath and Summerfield (1985) reported a study in which the intelligibility of audiovisual sentences presented in white noise deteriorated at much lower visual leads (160 ms; see also Pandey et al., 1986; Steinmetz, 1996) than those observed in the studies of Dixon and Spitz, Conrey and Pisoni, and Grant and colleagues. Based on these results, it would appear as though the perception of a continuous audiovisual speech signal remains intelligible across a wide range of signal delays (auditory or visual). It is not clear, however, what the exact interval range is since a high level of variability has been observed between the various studies that have been conducted to date (see Figure 1A).

In addition to the studies that have used continuous speech stimuli (i.e., passages, sentences), audiovisual temporal perception has also been evaluated for brief speech tokens using the McGurk effect (i.e., the visual influence on the perception of audiovisual speech; McGurk and MacDonald, 1976). For instance, Massaro et al. (1996) evaluated the temporal perception of consonant-vowel (CV) syllables under a wide range of different asynchronies using the fuzzy logic model of perception (FLMP). They found that audiovisual integration (as assessed by participants' reports of what was heard; i.e., speech identification task) was unaffected for auditory leads and lags of up to 250 ms (see also Massaro and Cohen, 1993). However, Munhall et al. (1996) reported results that were quite different. They presented vowel-consonant-vowel (VCV) stimuli and their results demonstrated that participants experienced the McGurk effect for auditory leads of 60 ms and lags of 240 ms. These values are similar to those that have been reported by Van Wassenhove et al. (2003, 2007) for CV stimuli (auditory leads from about 30 ms and lags of up to 200 ms; see Figure 1B, for a summary of these and other findings).

On the whole, the results of previous studies concerning the temporal perception of audiovisual speech stimuli have demonstrated that the audiovisual intelligibility of the speech signal remains high over a wide range of audiovisual temporal asynchronies. That said, this time-range (i.e., window) exhibits great variability across different studies (see Figure 1). This marked variation led us to investigate the possible factors that may affect the temporal perception of audiovisual speech (see Vatakis and Spence, 2006a–c, 2007, 2008, 2010). One factor that may help to explain the presence of variability in the temporal windows of multisensory integration previously observed for audiovisual speech stimuli relates to the particular speech stimuli utilized in the various studies. Specifically, the temporal window of integration for audiovisual speech has, in recent years, been shown to vary as a function of the physical parameters of the visual stimulus (e.g., inversion of the visual-speech stimulus promotes a wider window of integration; e.g., Vatakis and Spence, 2008) and the type of speech stimulus used (e.g., Vatakis and Spence, 2006b, 2007). Additionally, the temporal window of audiovisual integration appears to be wider for more complex stimuli (or more highly temporally correlated stimuli; e.g., syllables vs. words or sentences) than for simpler stimuli (such as light flashes and sound bursts; e.g., Navarra et al., 2005).

The present study focused on another possible factor that may affect the temporal perception of audiovisual speech, which is the effect that the physical changes due to the articulation of consonants (mainly characterized by the articulatory features of the place and manner of articulation and voicing; see Kent, 1997) and vowels (mainly characterized by the articulatory features of the height/backness of the tongue and roundedness of the lips; see Kent, 1997) may have on the parameters defining the temporal window for audiovisual integration. The optimal perception of speech stimuli requires the synergistic integration of auditory and visual inputs. However, according to the “information reliability hypothesis” in multisensory perception (whereby, the perception of a feature is dominated by the modality that provides the most reliable information), one could argue that the perception of a given speech token may, in certain cases, be dominated by the auditory-speech or the visual lip-movement that is more informative (e.g., Schwartz et al., 1998; Wada et al., 2003; Andersen et al., 2004; Traunmüller and Öhrström, 2007). Specifically, previous research has shown that auditory inputs are closely associated with the accurate detection of the manner of articulation and voicing of consonants, and the height/backness of vowels. Visual input, by contrast, provides essential cues regarding the accurate detection of the place of articulation of consonants and the roundedness of vowels (e.g., Miller and Nicely, 1955; Massaro and Cohen, 1993; Robert-Ribes et al., 1998; Girin et al., 2001; Mattys et al., 2002; Traunmüller and Öhrström, 2007). For example, Binnie et al. (1974) examine people's ability to identify speech by modulating the unimodal and bimodal contribution of vision and audition to speech using 16 CV syllables presented under noisy listening conditions. Their results indicated a large visual contribution to audiovisual speech perception (e.g., 41.4% visual dominance at −18 dB S/N), with the visual contribution being highly associated with the place of articulation of the CV syllables used. However, masking the auditory input has been shown to lead to a loss of information about the place of articulation, whereas information about the manner of articulation appears to be resistant to such masking (i.e., McGurk and MacDonald, 1976; Mills and Thiem, 1980; Summerfield and McGrath, 1984; Massaro and Cohen, 1993; Robert-Ribes et al., 1998; see Dodd, 1977, for a related study using CVCs; and Summerfield, 1983, for a study using vowels instead).

Previous studies of the effects of audiovisual asynchrony on speech perception have only been tested using a small number of syllables (e.g., Van Wassenhove et al., 2003; Vatakis and Spence, 2006b). It has therefore not been possible, on the basis of the results of such studies, to draw any detailed conclusions regarding the possible interactions of physical differences in speech articulation with audiovisual temporal perception. Additionally, given new findings indicating that high signal reliability leads to smaller temporal order thresholds (i.e., smaller thresholds imply high auditory- and visual-signal reliability; see Ley et al., 2009), further study of the temporal window of integration in audiovisual speech is necessary in order to possibly resolve the differences noted in previous studies. In the present study, we utilized a variety of different consonants (Experiments 1 and 2) and vowels (Experiment 3) in order to examine the possible effects that physical differences in articulation may have on the temporal perception of audiovisual speech stimuli. The stimuli used were selected according to the categorization of articulatory features established by the International Phonetic Alphabet (IPA) and they were sampled in such a way as to allow for comparison within and across different categories of articulation. We conducted a series of three experiments that focused on different articulatory features, and thus on the differential contribution of the visual- and auditory-speech signal. Specifically, in Experiment 1 (A–C), we focused on the place of articulation (i.e., the location in the vocal tract where the obstruction takes place; e.g., /p/ vs. /k/) and voicing features (i.e., the manner of vibration of the vocal folds; e.g., /p/ vs. /b/); in Experiment 2 (A–C), we looked at the manner of articulation (i.e., how the obstruction is made and the sound produced; e.g., /s/ vs. /t/); and, in Experiment 3, we explored the temporal perception of audiovisual speech as a function of the height/backness of the tongue and roundedness of the lips.

The temporal perception of the speech stimuli utilized in the present study was assessed using an audiovisual temporal order judgment (TOJ) task with a range of stimulus onset asynchronies (SOAs) using the method of constant stimuli (e.g., Spence et al., 2001). The TOJ task required the participants to decide on each trial whether the auditory-speech or the visual-speech stream had been presented first. Using the TOJ task permitted us to obtain two indices: the Just Noticeable Difference (JND) and the Point of Subjective Simultaneity (PSS). The JND provides a measure of the sensitivity with which participants could judge the temporal order of the auditory- and visual-speech streams. The PSS provides an estimate of the time interval by which the speech event in one sensory modality had to lead the speech event in the other modality in order for synchrony to be perceived (or rather, for the “visual-speech first” and “auditory-speech first” responses to be chosen equally often).

Overall, we expected that for the speech stimuli tested here (see Table 1) visual leads would be required for the synchrony of the auditory- and visual-signals to be perceived (i.e., PSS; except for the case of vowels, where auditory leads have been observed previously; Vatakis and Spence, 2006a). That is, during speech perception, people have access to visual information concerning the place of articulation before they have the relevant auditory information (e.g., Munhall et al., 1996). In part, this is due to the significant visual motion (e.g., the movement of facial muscles) that occurs prior to the auditory onset of a given syllable. In addition, according to the “information reliability hypothesis” (e.g., Schwartz et al., 1998; Traunmüller and Öhrström, 2007), we would expect that participants' TOJ responses would be differentially affected as a function of the “weight” placed on the auditory-speech or the visual lip-movement for the accurate detection of a particular speech token. That is, in the cases where the visual-speech signal is more salient (such as, for determining the place of articulation of consonants and the roundedness of vowels; such as, stimuli that involve high visible contrast with highly visible lip-movements; e.g., bilabial stimuli or rounded vowels; Experiments 1 and 3), we would expect participants' to be more sensitive to the presence of asynchrony (i.e., they should exhibit lower JNDs) as compared to less salient stimuli (such as, those involving tongue movement, as tongue movements are not always visible; e.g., as in the case of velar stimuli and unrounded vowels). No such effects would be expected for those cases where the auditory-speech input is more salient, such as, in the cases where the manner of articulation and voicing of consonants and the height/backness of vowels are evaluated (see Experiments 2 and 3). One must note, however, that in case auditory and visual signals are equally reliable, this should lead to smaller temporal order thresholds (i.e., JNDs; see Ley et al., 2009).

**Table 1:** **The main articulatory features used to categorize the consonant and vowel stimuli used in Experiments 1–3, as a function of: (A) the place of articulation, (B) the manner of articulation, and (C) the height and backness of the tongue and roundedness of the lips in Experiments 1–3**.

**Place of articulation**	**Manner of articulation**		
	**Experiment 1A**	**Experiment 1B**	**Experiment 1C**
	**Stop**	**Fricative**	**Nasal**
**(A) CONSONANTS**			
Bilabial	/b, p/	–	/m/
Labiodental	–	/v, f/	–
Dental	–	/ð, θ/	–
Alveolar	/d, t/	/z, s/	/n/
Velar	/g, k/	–	/η/
**Manner of articulation**	**Place of articulation**		
	**Experiment 2A**	**Experiment 2B**	**Experiment 2C**
	**Bilabial**	**Alveolar**	**Postalveolar**
**(B) CONSONANTS**			
Stop	/b/	/d/	–
Fricative	–	/z/	/ʒ/
Nasal	/m/	/n/	–
Affricative	–	–	/ʤ/
Lateral approximant	–	/l/	/r/
Approximant	/w/	–	–
**Height of tongue**	**Backness/roundedness**		
	**Front/unrounded**	**Back/rounded**	
**(C) VOWELS**			
High	/i/	/u/	
Mid	/ε/	/ɔ/	
Low	/æ/	/ɒ/	

## Experiments 1–3

### Materials and methods

#### Participants

All of the participants were naïve as to the purpose of the study and all reported having normal or corrected-to-normal hearing and visual acuity. The experiments were performed in accordance with the ethical standards laid down in the 1990 Declaration of Helsinki, as well as the ethical guidelines laid down by the Department of Experimental Psychology, University of Oxford. Each experiment took approximately 50 min to complete.

#### Apparatus and materials

The experiment was conducted in a completely dark sound-attenuated booth. During the experiment, the participants were seated facing straight-ahead. The visual stream was presented on a 17-inch (43.18 cm) TFT color LCD monitor (SXGA 1240 × 1024 pixel resolution; 60-Hz refresh rate), placed at eye level, approximately 68 cm in front of the participants. The auditory stream was presented by means of two Packard Bell Flat Panel 050 PC loudspeakers, one placed 25.4 cm to either side of the center of the monitor (i.e., the auditory- and visual-speech stimuli were presented from the same spatial location). The audiovisual stimuli consisted of black-and-white video clips presented on a black background, using Presentation (Version 10.0; Neurobehavioral Systems, Inc., CA). The video clips (300 × 280-pixel, Cinepak Codec video compression, 16-bit Audio Sample Size, Average pitch and amplitude (in Hz): 160 and 43, for consonants; 125 and 44, for vowels, respectively; 24-bit Video Sample Size, 30 frames/s) were processed using Adobe Premiere 6.0. The video clips consisted of the close-up views of the faces of a British male and two British females (visible from the chin to the top of the head), looking directly at the camera, and uttering a series of speech tokens (see Table 1). The open vowel /a/ was used for all of the articulated consonants in order to provide high levels of visible contrast relative to the closed mouth in the rest position. All of the audiovisual clips were 1400 and 2500 ms in duration (measured from the still frame before visual articulation of the speech token began to the last frame after articulation of the token had occurred) for consonants and vowels, respectively. All of the speech stimuli were recorded under the same conditions with the mouth starting and ending in a closed position. The articulation of all of the speech tokens was salient enough without our having to make the stimuli unnatural (i.e., by having the speakers exaggerate). In order to achieve accurate synchronization of the dubbed video clips, each original clip was re-encoded using XviD codec (single pass, quality mode of 100%).

At the beginning and end of each video clip, a still image and background acoustic noise was presented for a variable duration. The duration of the image and noise was unequal, with the difference in their duration being equivalent to the particular SOA tested (values reported below) in each condition. This aspect of the design ensured that the auditory and visual streams always started at the same time, thus ensuring that the participants were not cued as to the nature of the audiovisual delay with which they were being presented. In order to achieve a smooth transition at the start and end of each video clip, a 33.33 ms cross-fade was added between the still image and the video clip (Note here that a newer methodology by Maier et al., 2011, allows for better control and, thus, more accurate measurement of the synchrony of the audiovisual stimulus presentation). The participants responded using a standard computer mouse, which they held with both hands, using their right thumb for “visual-speech first” responses and their left thumb for “speech-sound first” responses (or vice versa, the response buttons were counterbalanced across participants).

#### Design

Nine SOAs between the auditory and visual streams were used: ±300, ±200, ±133, ±66, and 0 ms (the negative sign indicates that the auditory stream was presented first, whereas the positive sign indicates that the visual stream was presented first). This particular range of SOAs was selected on the basis of previous research showing that people can typically discriminate the temporal order of briefly-presented audiovisual speech stimuli at 75% correct at SOAs of approximately 80 ms (e.g., McGrath and Summerfield, 1985; Vatakis and Spence, 2006a; see also Munhall and Vatikiotis-Bateson, 2004). The participants completed one block of practice trials before the main experimental session in order to familiarize themselves with the task and the video clips. The practice trials were followed by five blocks of experimental trials. Each block consisted of two presentations of each of the stimuli used at each of the nine SOAs (presented in a random order using the method of constant stimuli; see Spence et al., 2001).

#### Procedure

At the start of the experiment, the participants were informed that they would have to decide on each trial whether the auditory-speech or visual-speech stream appeared to have been presented first. They were informed that they would sometimes find this discrimination difficult, in which case they should make an informed guess as to the order of stimulus presentation. The participants were also informed that the task was self-paced, and that they should only respond when confident of their response. The participants were informed that they did not have to wait until the video clip had finished before making their response, but that a response had to be made before the experiment would advance on to the next trial. The participants were instructed prior to the experiment not to move their heads and to maintain their fixation on the center of the monitor throughout each block of trials.

### Analysis

The proportions of “visual-speech first” responses at each SOA were converted to their equivalent z-scores under the assumption of a cumulative normal distribution (Finney, 1964). The data of each participant and condition from the seven intermediate SOAs (±200, ±133, ±66, and 0 ms) were cumulated and converted in z-scores to be fitted with a straight line (values were limited between 0.1 and 0.9; 0 and 1 were weighted using ((*n* − (*n* − 1))/*n*)^*^100 and ((*n* − 1)/*n*)^*^100), respectively, where *n* is the number of trials). Slope values were used to calculate the JND (JND = 0.675/slope; since ± 0.675 represents the 75% and 25% point on the cumulative normal distribution) and intercepts were used to obtain PSSs (PSS = −intercept/slope; see Coren et al., 2004, for further details). The ±300 ms points were excluded from this computation due to the fact that most participants performed near-perfectly at this interval and therefore these data points did not provide significant information regarding our experimental manipulations (cf. Spence et al., 2001, for a similar approach). For all of the analyses reported here, repeated measures analysis of variance (ANOVA) and Bonferroni-corrected *t*-tests (where *p* < 0.05 prior to correction) were used.

Preliminary analysis of the JND and PSS data using a repeated measures ANOVA revealed no effects^1^ attributable to the different speakers used to create the audiovisual stimuli, thus we combined the data from the three different speakers in order to simplify the statistical analysis (see Conrey and Gold, 2000, for a discussion of the effects of speaker differences on performance). The goodness of the data fits was significant for all conditions in all experiments conducted and the normality tests were also significant for all factors tested.

### Audiovisual physical signal saliency analysis

Bottom-up attention or saliency is based on the sensory cues of a stimulus captured by its signal-level properties, such as spatial, temporal, and spectral contrast, complexity, scale, etc. Similar to competitive selection, saliency can be attributed on the feature level, the stream level or the modality level. Based on perceptual and computational attention modeling studies, efficient bottom-up models and signal analysis algorithms have been developed by Evangelopoulos et al. (2008) in order to measure the saliencies of both the auditory and visual streams in audiovisual videos of complex stimuli such as movie video clips. These saliencies can be integrated into a multimodal attention curve, in which the presence of salient events is signified by geometrical features such as local extrema and sharp transition points. By using level sets of this fused audiovisual attentional curve, a movie summarization algorithm was proposed and evaluated.

**Figure 2:**
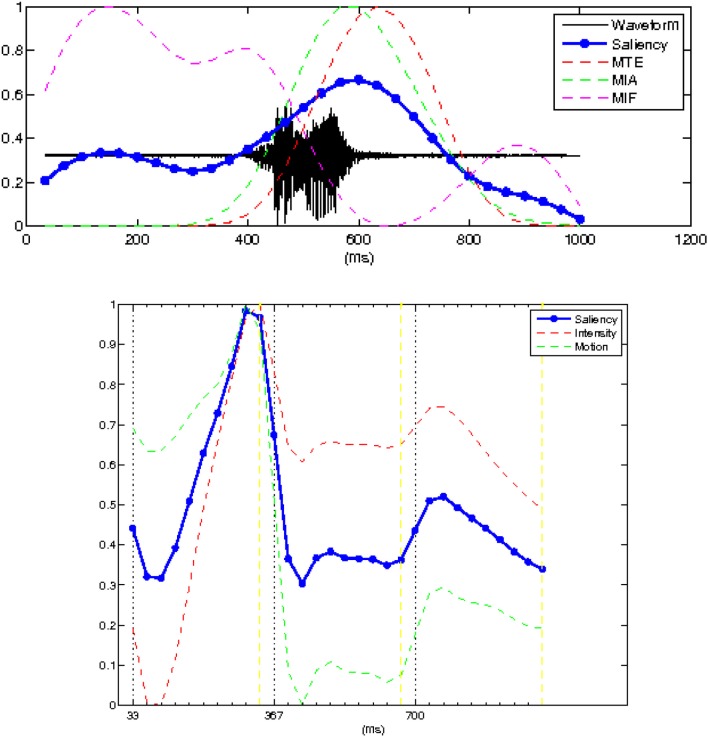
**Top panel** shows the acoustic waveform (solid black line) of the speech utterance with the auditory salience superimposed (thick solid line). The superimposed dashed lines show the temporal evolution of the three auditory cues (mean instantaneous energy, MTE, amplitude, MIA, and the frequency of the dominant frequency channel, MIF) whose linear combination gives the saliency. **Bottom panel** shows the visual saliency curve (in thick solid line). The superimposed dash lines shows the two visual cues that contributed to the computation of the visual saliency.

In the present study, we used the algorithms developed by Evangelopoulos et al. (2008) to separately compute two temporal curves indicating the saliencies of the auditory and visual streams for the stimuli presented (see Figure 2 for an example). Auditory saliency was captured by bandpass filtering the acoustic signal into multiple frequency bands, modeling each bandpass component as a modulated sinusoid, and extracting features such as its instantaneous amplitude and frequency. These features were motivated by biological observations and psychophysical evidence that, modulated carriers seem more salient perceptually to human observers compared to stationary signals (e.g., Tsingos et al., 2004; Kayser et al., 2005). In our experiments, the audio signal is sampled at 16 kHz and the audio analysis frames usually vary between 10 and 25 ms. The auditory filterbank consists of symmetric zero-phase Gabor filters, which do not introduce any delays. In the frequency domain, the filters are linearly arranged in frequency steps of 200–400 Hz, yielding a tessellation of 20–40 filters (details of the auditory feature extraction process can be found in Evangelopoulos and Maragos, 2006, and Evangelopoulos et al., 2008). The final auditory saliency temporal curve was computed as a weighted linear combination of three acoustic features: the mean instantaneous energy of the most active filter and the mean instantaneous amplitude, and frequency of the output from this dominant filter.

The visual saliency computation module is based on the notion of a centralized saliency map (Koch and Ullman, 1985; Itti et al., 1998) computed through a feature competition scheme, which is motivated by the experimental evidence of a biological counterpart in the human visual system (interaction/competition among the different visual pathways related to motion/depth and gestalt/depth/color, respectively; Kandel et al., 2000). Thus, visual saliency was measured by means of this spatiotemporal attentional model, driven by three feature cues: intensity, color (this feature was not used in our experiments given that the videos were presented in black and white), and motion. The spatiotemporal video volume (with time being the third dimension) was decomposed into a set of feature volumes, at multiple spatiotemporal scales (details on the visual feature extraction process can be found in Rapantzikos et al., 2009). By averaging over spatiotemporal neighborhoods, the feature (intensity and motion) volumes yielded a visual attentional curve whose value at each time instant represents the overall visual saliency of the corresponding video frame. The visual feature extraction process was synchronized with the respective auditory task on a frame-by-frame basis.

## Results and discussion

### Place of articulation and voicing for stop consonants (Experiment 1A)

In Experiment 1A, we evaluated whether (and how) the place of articulation and voicing of stop consonants (the manner of articulation was constant) influenced audiovisual TOJs. We categorized the data according to the factors of Place of articulation (three levels: bilabial, /b, p/; alveolar, /d, t/; velar, /g, k/) and Voicing (two levels: voiced, /b, d, g/; unvoiced, /p, t, k/; see Table 1A).

**Figure 3:**
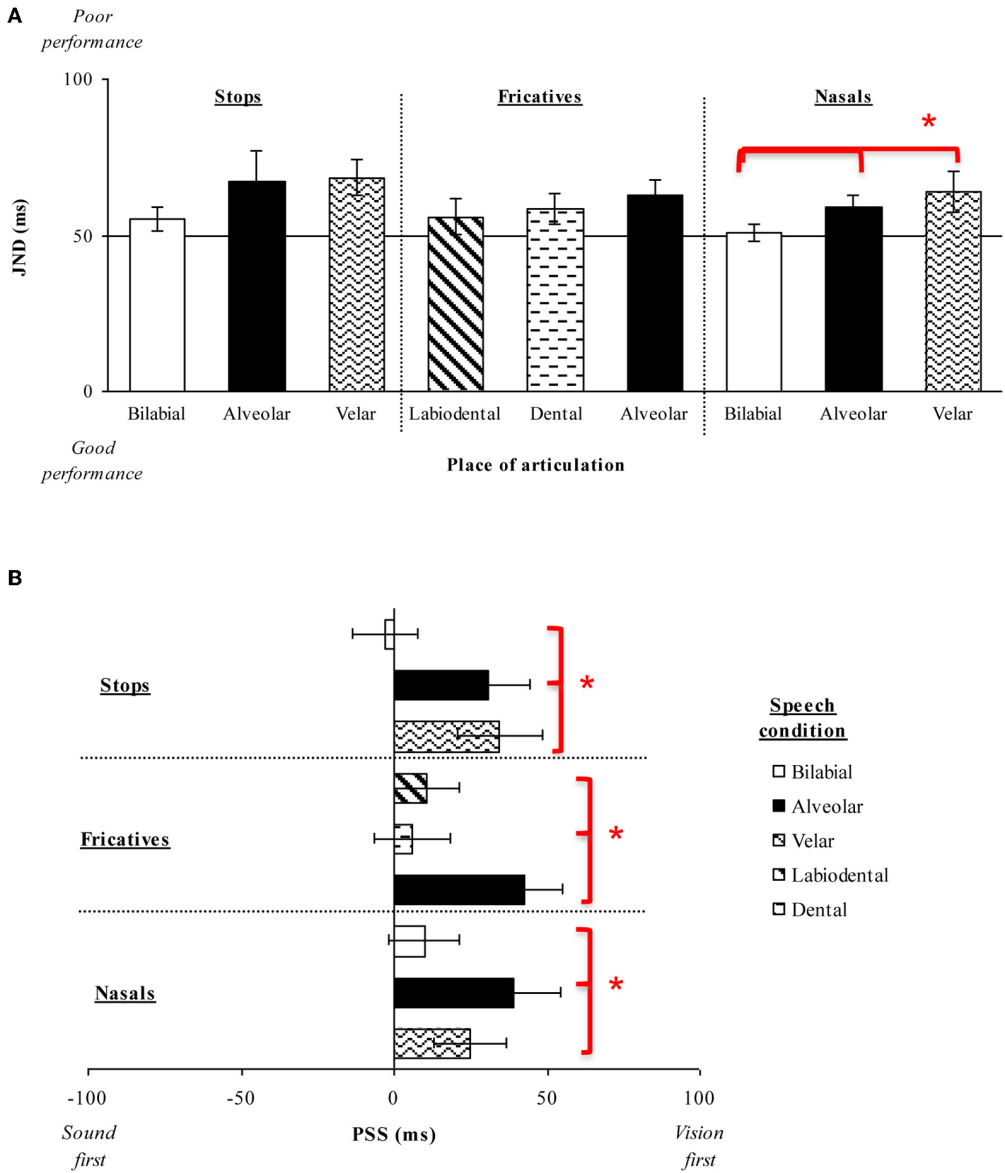
**(A)** Average JNDs and **(B)** PSSs for the place of articulation of the consonant stimuli presented in Experiment 1. The error bars represent the standard errors of the mean. Asterisks indicate significant differences between the various stimuli presented.

Fourteen participants (12 female; native English speakers) aged between 18 and 30 years (mean age of 24 years) took part in this experiment. A repeated measures ANOVA on the JND data revealed no significant main effect of Place of articulation [*F*_(2, 26)_ = 2.10, *p* = 0.15]. Although the participants were, numerically-speaking, more sensitive to the temporal order of the auditory- and visual-speech streams for bilabial stimuli (M = 55 ms) than for either alveolar (M = 67 ms) or velar (M = 68 ms) stimuli (see Figure 3A), this difference failed to reach statistical significance. There was also no significant main effect of Voicing [*F*_(1, 13)_ < 1, *n*.*s*.] (voiced, M = 64 ms; unvoiced, M = 63 ms; see Figure 6C), and the Place of Articulation by Voicing interaction was not significant either [*F*_(2, 26)_ = 1.27, *p* = 0.30].

The analysis of the PSS data revealed a significant main effect of Place of articulation [*F*_(2, 26)_ = 6.72, *p* < 0.01]. Large visual leads were required for the alveolar (M = 31 ms) and velar stimuli (M = 35 ms) as compared to the small auditory lead required for the bilabial (M = 3 ms) stimuli in order for the PSS to be reached (*p* < 0.05, for both comparisons; see Figure 3B). These results suggest that auditory leads were required when a visible place contrast was present for bilabial speech stimuli as compared to the large visual leads required for the invisible place contrast present in alveolar and velar stimuli (e.g., Girin et al., 2001). We also obtained a significant main effect of Voicing [*F*_(1, 13)_ = 12.65, *p* < 0.01], with voiced stimuli (M = 32 ms) requiring larger visual leads than unvoiced stimuli (M = 10 ms; see Figure 6D). There was no interaction between Place of articulation and Voicing [*F*_(2, 26)_ < 1, *n*.*s*.].

### Place of articulation and voicing for fricative consonants (Experiment 1B)

We further investigated the influence of the place of articulation and voicing on audiovisual temporal perception by testing fricative consonants. The data were categorized by the factors of Place of articulation (three levels: labiodental, /v, f/; dental, /ð, θ/; alveolar, /z, s/) and Voicing (two levels: voiced, /v, ð, z/; unvoiced, /f, θ, s/).

Fourteen new participants (10 female; native English speakers) aged between 18 and 34 years (mean age of 24 years) took part in this experiment. Analysis of the JND data revealed no significant main effect of Place of articulation [*F*_(2, 26)_ = 1.40, *p* = 0.26] or Voicing [*F*_(1, 13)_ = 3.74, *p* = 0.10], nor any interaction between these two factors [*F*_(2, 26)_ < 1, *n*.*s*.]. Participants' performance was very similar across the speech groups compared as a function of the Place of articulation and across the Voicing groups tested (i.e., Place of articulation: labiodental, M = 56 ms; dental, M = 58 ms; alveolar, M = 63 ms; Voicing: voiced, M = 57 ms; unvoiced, M = 61 ms; see Figures 3A,6C). Labiodental and dental stimuli are considered to be higher in visibility than alveolar stimuli (e.g., Binnie et al., 1974; Dodd, 1977; Cosi and Caldognetto, 1996). The JND values showed a trend toward higher visibility stimuli resulting in numerically smaller JNDs, however, this effect was not significant.

Analysis of the PSS data, however, revealed a significant main effect of Place of articulation [*F*_(2, 26)_ = 8.51, *p* < 0.01], with larger visual leads being required for the alveolar stimuli (M = 42 ms) than for the labiodental (M = 11 ms) or dental (M = 6 ms) stimuli (*p* < 0.01, for both comparisons; see Figure 3B). Given that labiodental and dental stimuli are considered to be higher in visibility than alveolar stimuli, the larger visual leads required for the alveolar stimuli provide similar results to those observed for the stop consonants tested earlier (Experiment 1A). There was no significant main effect for Voicing [*F*_(1, 13)_ < 1, *n*.*s*.] (see Figure 6D), nor was there any interaction between Place of articulation and Voicing [*F*_(2, 26)_ = 2.84, *p* = 0.10].

### Place of articulation for nasals (Experiment 1C)

Finally, we evaluated the influence of the Place of articulation on audiovisual TOJs by testing nasal consonants (the voicing factor was not evaluated since nasals are voiced-only). The data were evaluated according to Place of articulation (three levels: bilabial, /m/; alveolar, /n/; velar, /η/).

Thirteen new participants (nine female; native English speakers) aged between 19 and 34 years (mean age of 24 years) took part in the experiment. The analysis of the JND data resulted in a significant main effect of Place of articulation [*F*_(2, 24)_ = 4.45, *p* < 0.05], indicating that the participants were significantly more sensitive to the temporal order of the auditory- and visual-speech streams when evaluating bilabial stimuli (M = 51 ms) than when judging either alveolar (M = 60 ms) or velar (M = 64 ms) stimuli (*p* < 0.05 for both comparisons; see Figure 3A). These results are similar to the trend observed in Experiments 1A and B, with participants being more sensitive to the temporal order of the highly-visible speech tokens (e.g., Binnie et al., 1974; Sams et al., 1991; Robert-Ribes et al., 1998; Girin et al., 2001; see Massaro and Cohen, 1993, for evidence that people are better at identifying the syllable /ba/ as compared to the syllable /da/).

Analysis of the PSS data revealed a significant main effect of Place of articulation [*F*_(2, 24)_ = 2.62, *p* < 0.05], with the visual stream having to lead by a larger interval for the alveolar (M = 39 ms) and velar stimuli (M = 25 ms) than for the bilabial (M = 10 ms) stimuli in order for the PSS to be reached (*p* < 0.05, for both comparisons; see Figure 3B). Once again, these results are similar to those obtained previously for the stop consonants (Experiment 1A), where alveolar and velar stimuli were shown to require greater visual leads as compared to bilabial stimuli.

**Figure 4:**
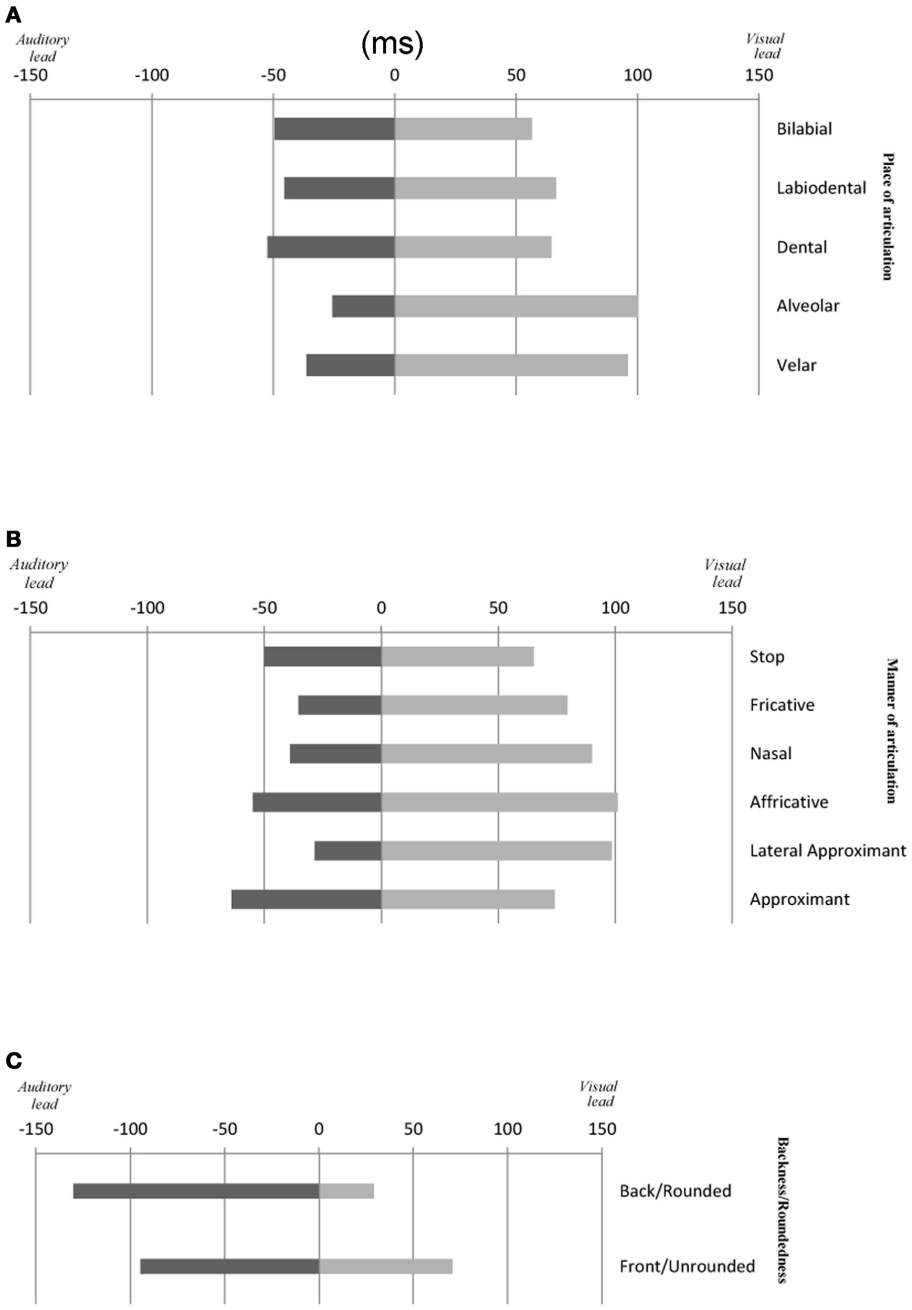
**Average temporal window of integration (PSS ± JND) for audiovisual speech as a function of: (A) the place of articulation and (B) the manner of articulation of consonant and (C) backness/roundedness of vowel stimuli used in this study**.

Overall, therefore, the results of Experiments 1A–C demonstrate that the visual signal had to lead the auditory signal in order for the PSS to be reached for the speech stimuli tested here (see Figure 3B). The sole exception was the bilabial stimuli in Experiment 1A, where an auditory lead of 3 ms was required (although, note that this value was not significantly different from 0 ms; [*t*_(13)_ < 1, *n*.*s*.]). These findings are supported by prior research showing that one of the major features of audiovisual speech stimuli is that the temporal onset of the visual-speech often occurs prior to the onset of the associated auditory-speech (i.e., Munhall et al., 1996; Lebib et al., 2003; Van Wassenhove et al., 2003, 2005). More importantly for present purposes, the results of Experiments 1A–C also revealed that the amount of time by which the visual-speech stream had to lead the auditory-speech stream in order for the PSS to be reached was smaller in the presence of a highly-visible speech stimulus (e.g., bilabials) than when the speech stimulus was less visible (e.g., as in the case of alveolars; see Figure 4A). This finding is also compatible with the cluster responses that are often reported in studies of speech intelligibility that have utilized McGurk syllables. For example, the presentation of a visual /ba/ together with an auditory /da/ often produces the response /bda/. This is not, however, the case for the presentation of a visual /da/ and an auditory /ba/ (i.e., where no /dba/ cluster is observed). This result can partially be accounted for by the faster processing of the visual /ba/ as compared to the visual /da/ (e.g., Massaro and Cohen, 1993). It should also be noted that the sensitivity of our participants' audiovisual TOJ responses was only found to differ as a function of changes in the place of articulation (a visually-dominant feature) in Experiment 1C but not in Experiments 1A–B. Additionally, no differences were obtained in participants' sensitivity as a function of voicing, which is an auditorily-dominant feature (e.g., Massaro and Cohen, 1993; Girin et al., 2001).

**Figure 5:**
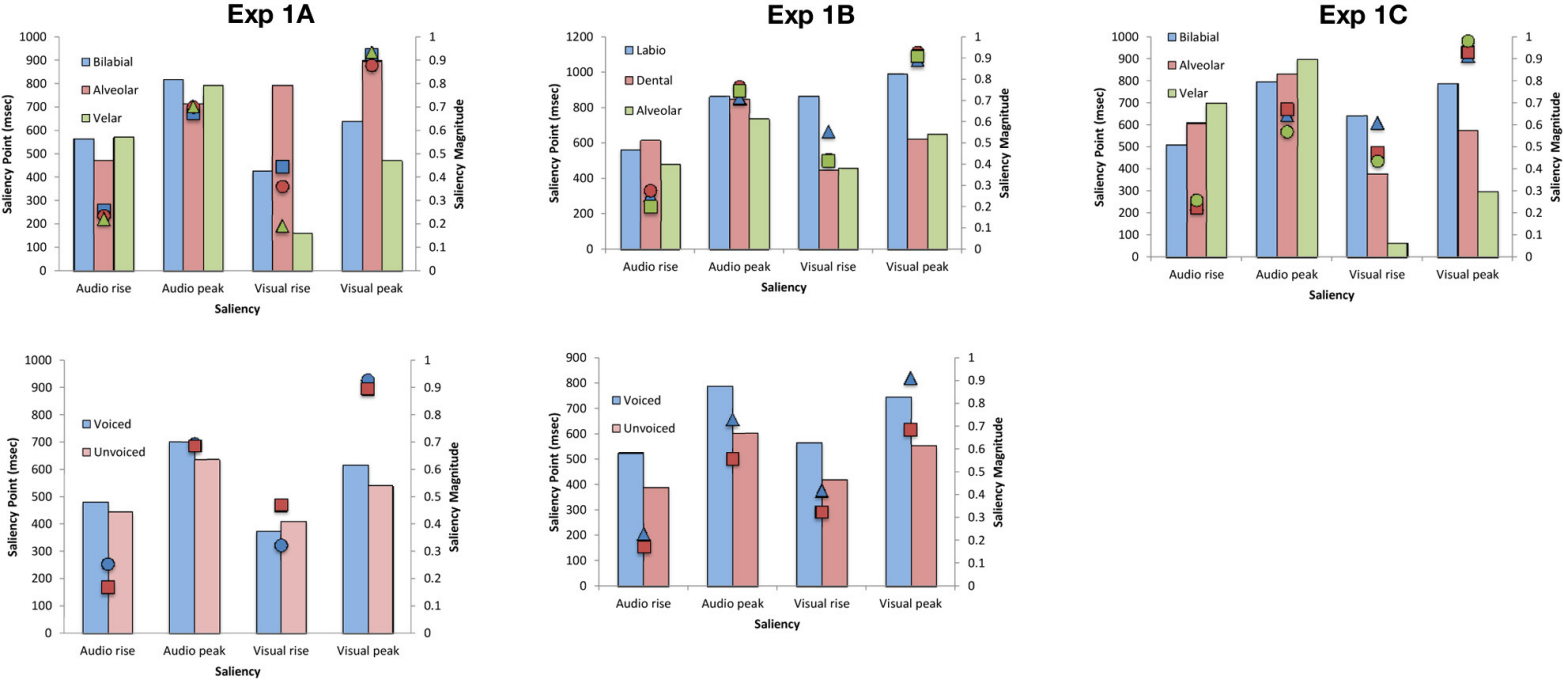
**Average saliency rise and peak (in ms) and saliency magnitude for each point for the audiovisual speech stimuli used in Experiments 1A–C as a function of the place of articulation and voicing**.

In order to examine the relationship between the perceptual findings described above and the physical properties of the audiovisual stimuli utilized in Experiments 1A–C, we conducted an auditory- and visual-saliency analysis of the synchronous audiovisual stimuli by using the computational algorithms developed by Evangelopoulos et al. (2008) to compute audio-visual saliencies in multimodal video summarization. The saliency analysis allowed calculation of the saliency rise (i.e., beginning of the saliency increase) and peak of each modality stream (in ms) and the magnitude of each saliency point (see Figure 5). In terms of the place of articulation, the saliency rise and peak occurred earlier for the visual stream as compared to the auditory stream for all stimuli except for the alveolar (Experiment 1A), labiodental (Experiment 1B), and bilabial (Experiment 1C) stimuli, where the reverse pattern was noted. The magnitude for each saliency rise and peak point, highlighted a clear trend for all stimuli with the magnitude being approximately the same for all points except for that of visual rise. Specifically, the highest saliency magnitude of visual rise was found for bilabials (Experiments 1A, C) and labiodentals (Experiment 1B).

Comparison of the physical and perceptual data revealed a trend whereby better TOJ performance coincided with visual rises that were larger in magnitude, thus, suggesting that higher in saliency stimuli lead to better detection of temporal order. In terms of PSS, the physical and perceptual data also exhibited a trend in terms of magnitude with larger visual leads being required for stimuli of lower magnitude (except for the case of dental stimuli in Experiment 1B), implying that lower magnitude stimulation is less salient, in terms of signal saliency, as compared to high in magnitude saliency points.

The saliency analysis for voicing did not reveal a consistent pattern, which might be due to the fact that voicing constitutes an auditorily-dominant feature. Specifically, the PSS-saliency magnitude pattern observed earlier was also present in Experiment 1A but not in 1B, where voiced stimuli were higher in magnitude in all saliency points.

The results of Experiments 1A–C therefore demonstrate that higher in saliency visual-speech stimuli lead to higher temporal discrimination sensitivity and smaller visual-stream leads for the speech signal. Previous studies support the view that visual-speech may act as a cue for the detection of speech sounds when the temporal onset of the speech signal is uncertain (e.g., Barker et al., 1998; Grant and Seitz, 1998, 2000; Arnold and Hill, 2001, though, see also Bernstein et al., 2004). Therefore, in the present study, it may be that the less visually salient speech stimuli required a greater visual lead in order to provide complementary information for the appropriate speech sound. We conducted a second set of experiments in order to explore how the manner of articulation of consonants affects audiovisual temporal perception. As mentioned already, the manner of articulation is an auditorily-dominant feature, thus we would not expect the visual-speech signal to modulate the temporal perception of consonants in the same manner as that observed in Experiment 1. The apparatus, stimuli, design, and procedure were exactly the same as in Experiment 1 with the sole exception that different groups of audiovisual speech stimuli were tested that now focused solely on the articulatory feature of the manner of articulation of consonants. All the stimuli tested in Experiments 2A–C were composed of voiced consonants with a constant place of articulation (see Table 1B).

### Manner of articulation for bilabials (Experiment 2A)

We were interested in the influence that the manner of articulation of voiced bilabials has on the temporal aspects of audiovisual speech perception. We categorized the data according to the factor of Manner of articulation (three levels: stop, /b/; nasal, /m/; and approximant, /w/).

**Figure 6:**
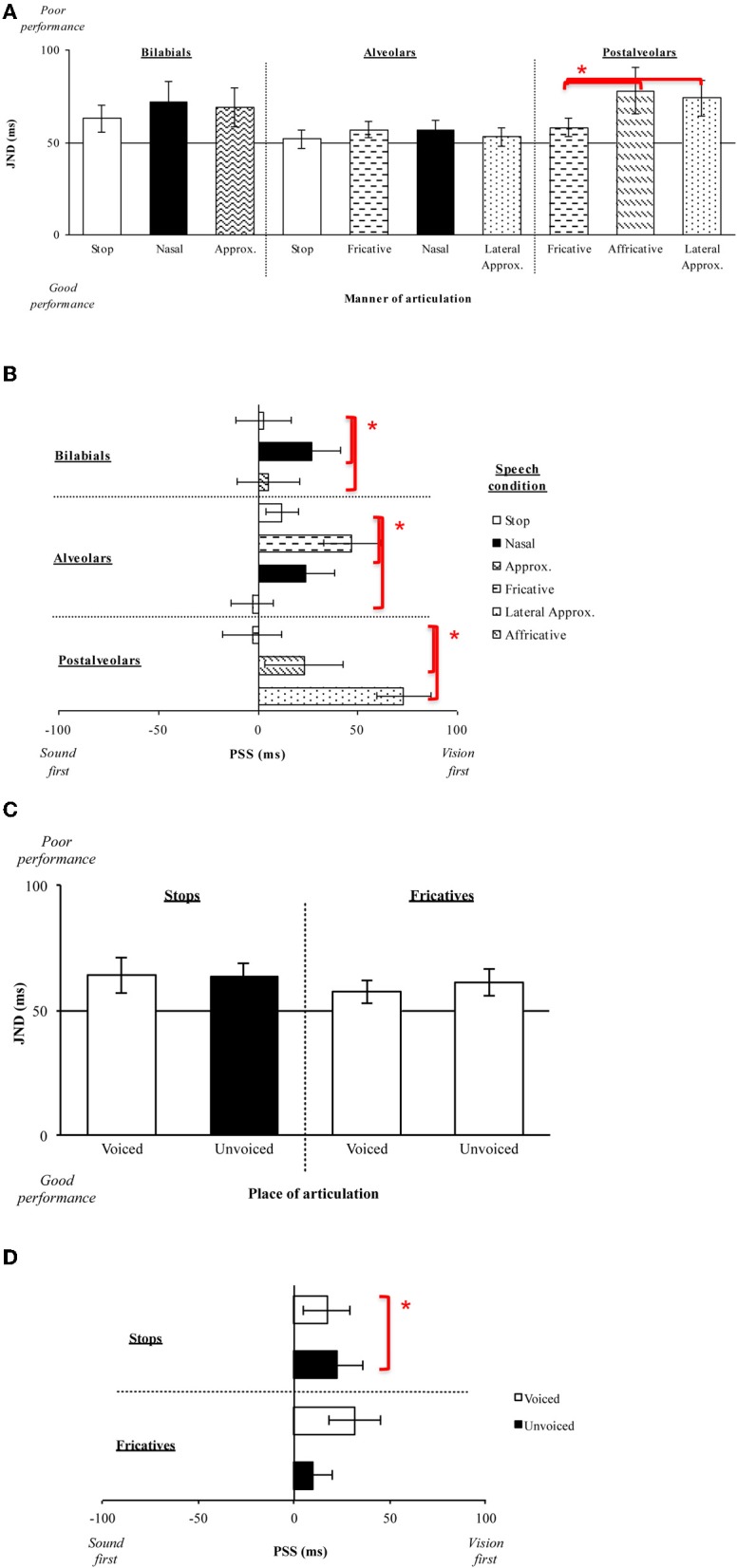
**(A)** Average JNDs and **(B)** PSSs for the manner of articulation of the consonant stimuli presented in Experiment 2. The error bars represent the standard errors of the mean. Asterisks indicate significant differences between the various stimuli presented. **(C)** Average JNDs and **(D)** PSSs for the voicing of the stimuli presented in Experiment 1.

Eleven new participants (six female; native English speakers) aged between 18 and 30 years (mean age of 24 years) took part in the experiment. The participants were numerically somewhat more sensitive to the temporal order of the stop (Mean JND = 63 ms) and approximant (M = 69 ms) stimuli than for the nasal stimuli (M = 72 ms), although the main effect of the Manner of articulation was not significant [(*F*_(2, 20)_ < 1, *n*.*s*.); see Figure 6A]. The analysis of the PSS data, however, revealed a significant main effect of the Manner of articulation (*F*_(2, 20)_ = 5.92, *p* < 0.05), with significantly larger visual leads being required for the nasal stimuli (M = 27 ms) in order for the PSS to be reached as compared to the much smaller visual leads required for the stop (M = 3 ms) and approximant (M = 5 ms) stimuli (*p* < 0.05, for both comparisons; see Figure 6B). The results obtained here are similar to those reported in Experiments 1A–C in terms of the PSS data, where significantly smaller visual leads were required for the highly-visible stop and approximant stimuli as compared to the less visible nasal stimuli.

### Manner of articulation for alveolars (Experiment 2B)

We were also interested in what role, if any, the manner of articulation of voiced alveolars would play in the temporal aspects of audiovisual speech perception. We evaluated the data based on the factor of Manner of articulation (four levels: stop, /d/; fricative, /z/; nasal, /n/; and lateral approximant, /l/).

Eleven new participants (six female; native English speakers) aged between 19 and 30 years (mean age of 24 years) took part in the experiment. The participants were slightly more sensitive to the temporal order of stop (M = 52 ms) and lateral approximant (M = 53 ms) stimuli than to the temporal order of the fricative (M = 57 ms) and nasal (M = 57 ms) stimuli (see Figure 6A). However, the analysis of the JND data revealed no significant main effect of the Manner of articulation [*F*_(3, 30)_ = 1.23, *p* = 0.32]. The analysis of the PSS data highlighted a significant main effect of the Manner of articulation [*F*_(3, 30)_ = 9.13, *p* < 0.01], with significantly larger visual leads being required for the fricative stimuli (M = 47 ms) as compared to the visual leads required for the stop stimuli (M = 12 ms), and the auditory leads required for the lateral approximant (M = 3 ms) stimuli (*p* < 0.05; *p* < 0.01, respectively).

### Manner of articulation for postalveolars (Experiment 2C)

Finally, we evaluated how the manner of articulation of voiced postalveolars influences the temporal aspects of audiovisual speech perception by varying the stimuli used as a function of the Manner of articulation (three levels: fricative, /ʒ; affricative, /ʤ; and lateral approximant, /r/).

Eleven new participants (five female; native English speakers) aged between 18 and 34 years (mean age of 24 years) took part in this experiment. The analysis of the JND data revealed a significant main effect of the Manner of articulation [*F*_(2, 20)_ = 4.60, *p* < 0.05], with the participants being significantly more sensitive to the temporal order of fricative stimuli (M = 58 ms) than of affricative (M = 78 ms) or lateral approximant stimuli (M = 74 ms; *p* < 0.05, for all comparisons; see Figure 6A). A similar analysis of the PSS data also revealed a significant main effect of the Manner of articulation [*F*_(2, 20)_ = 12.24, *p* < 0.01]. Fricative stimuli (M = 3 ms) required auditory leads for the PSS to be reached as compared to the visual leads required for the affricative (M = 23 ms) and lateral approximant (M = 73 ms) stimuli (*p* < 0.05, for all comparisons; see Figure 6B). The results obtained with the postalveolar stimuli tested here agree with the general findings of Experiment 1, whereby stimuli with a lower JND value (i.e., stimuli where participants are more sensitive to the temporal order of the presentation of the auditory and visual stimuli) also required smaller visual leads (i.e., fricatives). However, lateral approximant stimuli are generally considered to be more visible than fricative stimuli, therefore the higher sensitivity (in terms of the lower JNDs) observed here for fricative stimuli does not agree with the idea that highly-visible stimuli result in improved sensitivity to temporal order (i.e., lower JNDs).

**Figure 7:**
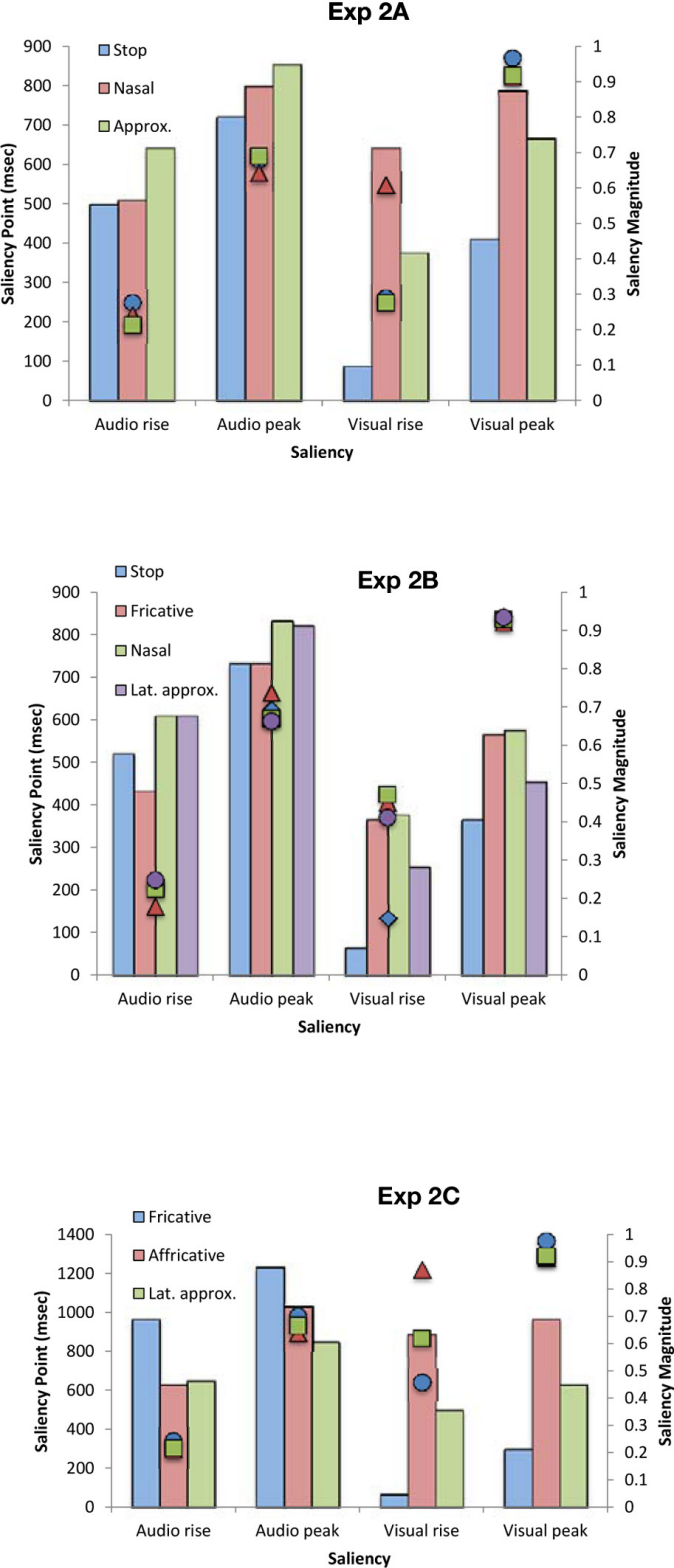
**Average saliency rise and peak (in ms) and saliency magnitude for each point for the audiovisual speech stimuli used in Experiments 2A–C as a function of the manner of articulation**.

The saliency analysis of the auditory and visual signals for the stimuli presented in Experiments 2A–C (see Figure 7) once again revealed saliency changes of greater magnitude for the points of visual rise, while the visual rise was not reached earlier as consistently as found in Experiment 1. Specifically, visual rise was earlier for stops and approximants in Experiment 2A, stop and lateral approximant in Experiment 2B, and fricative and lateral approximant in Experiment 2C. This earlier visual rise also coincides with the previously-noted trend toward better sensitivity to temporal order for these stimuli (which, however, only reached significance in the behavioral data for fricatives in Experiment 2C). In terms of saliency magnitude, no specific trend was observed (as with voicing in Experiment 1). This null result might be driven by the fact that the manner of articulation is an auditorily-driven feature. Specifically, in Experiments 2A and 2C, the participants required larger visual leads for nasals and affricatives, respectively, while physically those stimuli were higher in saliency magnitude for visual rise but saliency was reached earlier for auditory rise. Fricatives and lateral approximants in Experiments 2B and 2C, respectively, required perceptually visual leads for synchrony to be perceived, while the saliency magnitude was high and the saliency rise was reached earlier for the visual stream.

The results of Experiments 2A–C demonstrate similar results to those observed in Experiments 1A–C in terms of the PSS data. That is, the amount of time by which the visual-speech stream had to lead the auditory-speech stream in order for the PSS to be reached was smaller in the presence of highly-visible speech stimuli as compared to less-visible speech stimuli (see Figure 4B). There was no consistent pattern of the behavioral and the physical data, however, this result may be accounted for by the fact that the manner of articulation is a feature (just like voicing) that is highly associated with the auditory input (Massaro and Cohen, 1993; Girin et al., 2001). The results of Experiments 2A–C also revealed (just as had been highlighted in Experiments 1A–C) that the visual signal had to precede the auditory signal in order for the PSS to be achieved (except in the case of fricative and lateral approximant stimuli where a small auditory lead was observed; Experiments 2C and 2B, respectively; However, once again, this value was not significantly different from 0 ms; [*t*_(10)_ = 1.10, *p* = 0.32]; [*t*_(10)_ < 1, *n*.*s*.], respectively).

By themselves, the results of Experiments 2A–C suggest that visual-speech saliency influences the temporal perception of audiovisual speech signals mainly in terms of the PSS data. The perceptual and physical data do not, however, exhibit a consistent pattern. This may reflect the fact that the manner of articulation represents a feature that is largely dependent on the auditory signal for successful extraction of the speech signal, thus making the visible identification of all voiced consonants particularly difficult (due to the fact that neither the movements of the velum nor those of the vocal folds are visible; see Cosi and Caldognetto, 1996), thus supporting the “information reliability hypothesis” (e.g., Schwartz et al., 1998; Wada et al., 2003; Andersen et al., 2004; Traunmüller and Öhrström, 2007). The majority of previous research on speech perception has focused on the use of CV combinations as their main stimuli. In our third experiment, therefore, we further explored how physical differences in the articulation of vowels (in a non-consonant context) affect the temporal aspects of audiovisual speech perception. Here, we would expect that visual-speech should influence the JND data (in a similar way as that observed in Experiment 1) as a function of the roundedness of vowels, since this is the visually-dominant feature for vowels.

### Backness/roundedness and height for vowels (Experiment 3)

In our third and final experiment, we were interested in what role, if any, the backness/roundedness and height of articulation of vowels would play in the temporal aspects of audiovisual speech perception. The data were categorized according to the factors of Height (three levels: High, /i, u/; Mid, /ε, ɔ/; and Low, /æ, ɒ/) and Backness/Roundedness of articulation (two levels: front/unrounded, /i, ε, æ/ and back/rounded, /u, ɔ, ɒ/; see Table 1C).

**Figure 8:**
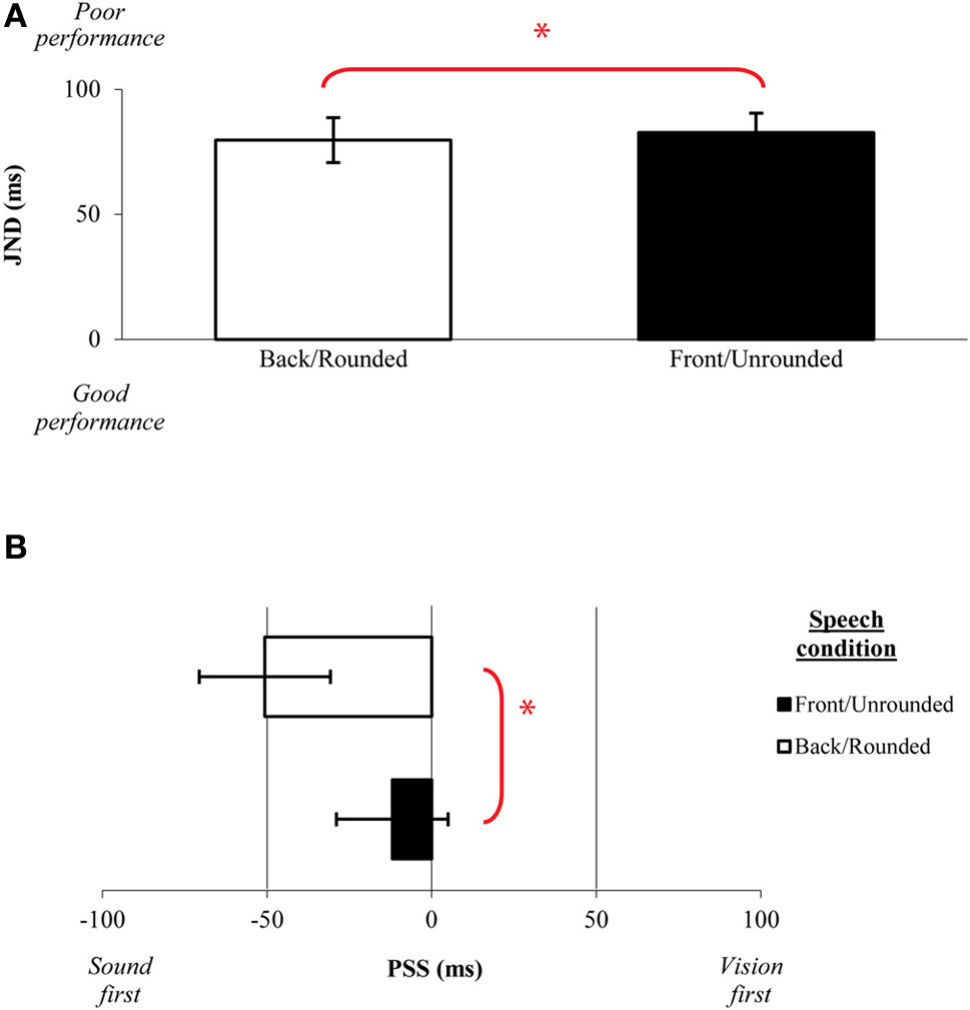
**Average (A) JNDs and (B) PSSs for the backness/roundedness of the vowel stimuli presented in Experiment 3.** The error bars represent the standard errors of the mean. Asterisks indicate significant differences between the various stimuli presented.

Eleven new participants (eight female; native English speakers) aged 19–30 years (mean age of 23 years) took part in this experiment. Analysis of the JND data revealed in a significant main effect of Backness/ Roundedness [*F*_(1, 10)_ = 4.75, *p* = 0.05], with participants' being significantly more sensitive to the temporal order of the audiovisual speech stimuli when judging back/rounded stimuli (M = 73 ms) as compared to front/unrounded stimuli (M = 89 ms; see Figure 8A). No significant main effect of Height was obtained [*F*_(2, 20)_ < 1, *n*.*s*.], nor any interaction between Height and Backness/Roundedness in vowels [*F*_(2, 20)_ < 1, *n*.*s*.]. A similar analysis of the PSS data revealed a significant main effect of Backness/Roundedness [*F*_(1, 10)_ = 18.60, *p* < 0.01], with larger auditory leads being required for rounded vowels articulated at the back of the tongue (M = 51 ms) than for unrounded vowels articulated at the front (M = 12 ms; see Figure 8B). The large auditory leads observed for Roundedness agrees with research showing that the recognition of rounded stimuli is difficult for both automatic speech recognition systems, with the systems being blind to roundedness, and humans who recruit more subtle physical cues and possibly more complex operations along the auditory pathway in perceiving rounded vowels (e.g., Eulitz and Obleser, 2007).

**Figure 9:**
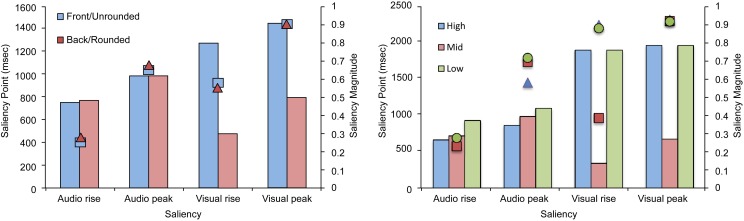
**Average saliency rise and peak (in ms) and saliency magnitude for each point for the audiovisual speech stimuli used in Experiment 3 as a function of the roundedness and height**.

The saliency analysis of the stimuli used in Experiment 3 (see Figure 9) showed a similar trend to that observed in Experiment 1. Specifically, the analysis revealed that, for back/rounded vowels, the saliency for both rise and peak was reached earlier for the visual stream and participants were better in their TOJ performance, the reverse pattern was observed for front/unrounded vowels. In terms of PSS, front/unrounded vowels were found to require large auditory leads with the saliency being noted earlier for the auditory stream (i.e., earlier auditory rise and peak) but was of lower magnitude (i.e., the highest magnitude was noted for the visual rise and peak). No specific trend was observed for height, a highly auditory feature (similar to Experiment 2).

Overall, the results of Experiment 3 replicate the patterns of JND and PSS results obtained in Experiments 1A–C and the PSS findings obtained in Experiments 2A–C. Specifically, larger auditory leads were observed for the highly salient rounded vowels as compared to the lower in saliency unrounded vowels (e.g., see Massaro and Cohen, 1993, for a comparison of /i/ and /u/ vowels and the /ui/ cluster; Traunmüller and Öhrström, 2007). Additionally, the participants were also more sensitive to the temporal order of the rounded vowels as compared to the unrounded vowels. It should, however, be noted that differences in the sensitivity to temporal order were only found as a function of roundedness/backness, while no such differences were observed as a function of the height of the tongue positions, which happens to be a highly auditory-dominant feature. The fact that auditory leads were required for all of the vowels tested here is consistent with similar findings reported previously by Vatakis and Spence (2006a).

## General discussion

The three set of experiments reported in the present study provide empirical evidence regarding how physical differences in the articulation of different speech stimuli can affect audiovisual temporal perception utilizing a range of different consonant and vowel stimuli. The speech stimuli used here were compatible (i.e., both the visual-speech and auditory-speech referred to the same speech event). This contrasts with the large number of previous studies of the relative contribution of audition and vision to speech perception that have utilized incompatible speech signals (as in the McGurk effect; McGurk and MacDonald, 1976). The stimuli were also presented in the absence of any acoustic noise. This was done in order to explore how participants weight differently the auditory and visual information in speech given that surely the system weights the reliability of the modality information even under quiet settings (e.g., Andersen et al., 2004). Additionally, we utilized speech stimuli from three different speakers, while the majority of previous studies have used different tokens uttered by the same speaker (e.g., see Conrey and Gold, 2000, for a discussion of this point). The use of different speakers strengthens the present study since it takes account of the possible variability that may be present during the articulation of speech tokens by different individuals. Additionally, an audiovisual saliency analysis of the stimuli was conducted in order to make comparisons between the physical signal data and the behavioral data collected. Taken together, the results of the experiments reported here demonstrate (but see Maier et al., 2011 for different control of stimulus synchronous presentation) that the onset of the visual-speech signal had to precede that of the onset of the auditory-speech for the PSS to be reached for all the consonant stimuli tested (see Lebib et al., 2003; Van Wassenhove et al., 2003, 2005).

We hypothesize that the results of the present study show evidence that integration is being dominated by the modality stream that provides the more salient information (e.g., place vs. manner of articulation of consonants; Schwartz et al., 1998; Wada et al., 2003). Our results also support the idea that the degree of saliency of the visual-speech signal can modulate the visual lead required for the two stimuli to be perceived as simultaneous. That is, the more visible (i.e., greater in saliency magnitude) the visual signal, the smaller the visual lead that is required for the PSS to be reached. These findings accord well with (Van Wassenhove et al.'s, 2005, p. 1183] statement that “… the more salient and predictable the visual input, the more the auditory processing is facilitated (or, the more visual and auditory information are redundant, the more facilitated auditory processing.”

Visual speech signals represent a valuable source of input for audiovisual speech perception (i.e., McGrath and Summerfield, 1985; Dodd and Campbell, 1987) that can influence the acoustic perception of speech in both noisy and quiet conditions (e.g., Dodd, 1977; Calvert et al., 1997; Barker et al., 1998; Arnold and Hill, 2001; Girin et al., 2001; Möttönen et al., 2002). The visual input can also reduce the temporal and spectral uncertainty of the speech signal by directing auditory attention to the speech signal (Grant and Seitz, 2000), and can, in certain cases, serve as a cue that facilitates the listener's ability to make predictions about the upcoming speech sound and assist in the successful extraction of the relevant auditory signal (see Barker et al., 1998; Van Wassenhove et al., 2003, 2005). The idea that the visual signal serves as a cue that may help to identify the auditory signal is supported by the results of Experiments 1 and 3, where the visual signal had to lead the auditory signal (even for the cases of manner of articulation and voicing where the auditory input has a dominance over visual input; Massaro and Cohen, 1993; Girin et al., 2001; Van Wassenhove et al., 2005) for synchrony to be perceived depending on the degree of saliency of the speech stimulus presented.

The complementarity of vision and audition in the case of speech perception is more evident in those cases where the phonetic elements that are less robust in the auditory domain (in the presence of auditory noise) are the ones that are the most salient in the visual domain (i.e., Binnie et al., 1974; Summerfield, 1979, 1983, 1987; Grant et al., 1985; Robert-Ribes et al., 1998; De Gelder and Bertelson, 2003). It appears that those speech features that are hardest to discern on the basis of their auditory input benefit most from the addition of the visual inputs and vice versa. According to our results, highly salient speech contrasts (such as bilabial stimuli) lead to relatively shorter processing latencies for the speech signal, while lower in saliency (i.e., less visible) visual inputs lead to longer processing latencies. These findings are supported by the results of imaging studies reported by Van Wassenhove et al. (2003, 2005). There it was argued that salient visual inputs (as in /pa/) affect auditory speech processing (at very early stages of processing: i.e., within 50–100 ms of stimulus onset) by enabling observers to make a prediction concerning the about-to-be-presented auditory input. Additional support for this conclusion comes from the results of a study by Grant and Greenberg (2001) in which the introduction of even small auditory leads (of as little as 40 ms) in the audiovisual speech signal resulted in a significant decline in speech intelligibility while intelligibility remained high when the visual signal led by as much as 200 ms.

Previous research on the topic of audiovisual synchrony perception has demonstrated that the human perceptual system can recalibrate to the temporal discrepancies introduced between auditory and visual signals and that this recalibration appears to vary as a function of the type of stimuli being presented (i.e., Navarra et al., 2005; Vatakis and Spence, 2007). It has been shown that when people are presented with simple transitory stimuli (such as, light flashes and sound bursts) smaller discrepancies between the temporal order of the two signals can be perceived (e.g., Hirsh and Sherrick, 1961; Zampini et al., 2003), as compared to more complex events (such as speech, object actions, or musical stimuli) where audiovisual asynchrony appears to be harder to detect (e.g., Dixon and Spitz, 1980; Grant et al., 2004; Navarra et al., 2005; Vatakis and Spence, 2006a,b). For instance, studies using simple audiovisual stimuli (such as, sound bursts and light flashes) have typically shown that auditory and visual signals need to be separated by approximately 60–70 ms in order for participants to be able to accurately judge which sensory modality was presented first (e.g., Zampini et al., 2003). While studies using more complex stimuli, such as audiovisual speech, have shown that the asynchrony of the audiovisual signals (i.e., visual- and auditory-speech) that can be tolerated can reach auditory leads of 100 ms or more, or auditory lags of at least 200 ms (e.g., Dixon and Spitz, 1980; Grant and Greenberg, 2001; Grant et al., 2004; Vatakis and Spence, 2006a,b, 2007). As discussed in the Introduction, the purported size of the temporal window of integration for audiovisual speech (a stimulus that is highly complex) exhibits great variability between published studies. The present findings highlight one important factor underlining this variability, which relates to the physical differences that are naturally present in the articulation of different consonants and vowels. The results of this study show that visual-speech has to lead auditory-speech in order for the two to be judged as synchronous, and the fact that larger visual lead times were required for lower saliency visual-speech signals, could provide one possible account for the human perceptual system's higher tolerance to asynchrony for the case of speech as compared to for simpler stimuli.

Overall, therefore, the results of the three sets of experiments reported here replicate previous findings that visual speech signals typically precede the onset of the speech sound signal in audiovisual speech perception (e.g., Munhall et al., 1996). In addition, our findings also extend previous research by showing that this precedence of the visual signal changes as a function of the physical characteristics in the articulation of the visual signal. That is, highly-salient visual-speech signals require less of a lead over auditory signals than visual-speech signals that are lower in saliency. Finally, our results support the analysis-by-synthesis model, whereby the precedence of the visual signal leads the speech-processing system to form a prediction regarding the auditory signal. This prediction is directly dependent on the saliency of the visual signal, with higher saliency signals resulting in a better prediction of the auditory signal (e.g., Van Wassenhove et al., 2005). It would be interesting in future research to explore how coarticulation cues affect the temporal relationship between auditory- and visual-speech signals observed in this study, since the oral and extra-ocular movements of a particular speech token are known to change depending on the context in which they are uttered (e.g., from syllable to word; Abry et al., 1994). In closing, future studies should further explore the relationship between the physical characteristics of the audiovisual speech signal (as explored by Chandrasekaran et al. (2009), for labial speech stimuli and in this manuscript in terms of saliency) and the behavioral data obtained in terms of temporal synchrony.

### Conflict of interest statement

The authors declare that the research was conducted in the absence of any commercial or financial relationships that could be construed as a potential conflict of interest.

## Footnotes

^1^Experiments 1A, B—no significant interaction between Place, Voicing, and Speaker in either the JND or PSS data [*F*_(4, 52)_ < 1, *n*.*s*.], for both; Experiment 1C—no significant interaction of Place and Speaker for the JND and PSS data; *F*_(4, 48)_ = 1.35, *p* = 0.27; *F*_(4, 48)_ = 2.44, *p* = 0.11, respectively; Experiments 2A, C—no significant interaction of Manner of articulation and Speaker for the JND and PSS data; *F*_(4, 40)_ < 1, *n*.*s*., for both; Experiment 2B—no significant interaction of Manner of articulation and Speaker for the JND and PSS data; *F*_(6, 60)_ = 1.15, *p* = 0.20; *F*_(6, 60)_ = 1.27, *p* = 0.28, respectively.
